# Adhesive Systems Used in Indirect Restorations Cementation: Review of the Literature

**DOI:** 10.3390/dj7030071

**Published:** 2019-07-01

**Authors:** Cristian Abad-Coronel, Belén Naranjo, Pamela Valdiviezo

**Affiliations:** Faculty of Dentistry, Universidad de Cuenca-Ecuador, Cuenca CP 010107, Ecuador

**Keywords:** dental adhesives, indirect restorations, cementation, bond strength

## Abstract

New technologies are changing the therapeutical options to do indirect restorations and new adhesive systems are continuously introduced to be used by clinicians. Different interactions between restorations, adhesive systems components, enamel and dentin require having criteria based on the selection of the adhesive system, ensuring the longevity of the restorations and the preservation of the biological remnant. The adhesion force to the dental tissue is one of the indicatives of the behavior of the adhesive systems and influences the behavior of the treatments with direct and indirect restorations. The objective of this search was to find the adhesive systems with the best results in terms of the adhesion strength of indirect restorations on the dental tissues. The search was conducted in two MEDLINE digital databases (PubMed), and the Cochrane Library with a search strategy based on the combination of MeSH (Medical Subject Headings) keywords. This systematic review used the PRISMA guide (Preferred Reporting Items for Systematic Reviews and Meta-Analysis). According to this review, the 3-step adhesive systems were the best performing and still are the gold standard for the cementing of indirect restorations. In addition, it can be concluded that self-etched adhesive systems reduce the time spent in clinical practice, however at the interface level they behave as permeable membranes more susceptible to degradation.

## 1. Introduction

Dental restorations are indicated to rehabilitate lost tissues for several reasons, such as the presence of decay, fractures, dental wear, loss of structure, functionality and aesthetics, as well as for the replacement of failed anterior restorations [1,2]. Indirect restorations allow greater control of form and function, especially in situations of severe loss of dental tissue. The materials used for this type of restorations can be ceramic, hybrid or polymeric. Ceramic is a widely used material since its physical and optical properties have great capacity to blend into the oral environment, fulfilling aesthetic and functional requirements. Its clinical behavior is similar or slightly better than resin restorations [3]. They are indicated for veneers, inlays, onlays and total coating restorations. Among its advantages are chemical stability, biocompatibility, low thermal conductivity, greater resistance to compression, translucency and fluorescence [4,5]. In general, conservative, minimally invasive preparations are used compared to conventional techniques and with less mechanical requirements in the preparation for retentive purposes. Therefore, the adhesive factor is a critical point in clinical success. Despite all advantages mentioned in the case of indirect restorations, the challenge is increased because there are more adhesive interfaces compared to direct restorations, namely: One interface on the surface of the tooth and a second on the surface of the restoration [6,7,8,9,10,11].

The adhesion process is achieved with resin cements through mechanical interlocking plus chemical bonding through silanization [12]. In addition, it requires multiple steps for the preparation of the tooth surface and the ceramic, being a sensitive technique susceptible to contamination and that also consumes time in clinical practice [13,14]. The correct application of the technique is the key to the success and longevity of the restorations [14,15,16,17]. The adequate selection of the adhesive system furthermore allows to take advantage of all the advantages offered by an indirect treatment [6,7,8,14,18,19,20]. For example, when the adhesive process is successful, the cementation allows a greater fracture resistance of the ceramic materials facilitated by the impregnation of the cement in the irregularities of the internal surface of the restoration and, in turn, promotes greater bonding strength and inhibits propagation of possible fissures [11,17,21,22].

In enamel, adhesion is very reliable. Enamel is formed by 94% to 96% inorganic substances, 1% to 4% water and 4% to 5% organic substances. It presents a greater intermolecular force and high surface energy. It is formed by prisms that extent from the amelodentinal junction to the external surface of the enamel. Application of the acid acts on the prisms generating three different patterns of etching. Pattern 1 acts at the core level of the prisms, pattern 2 acts at the periphery of the prisms and pattern 3 is a combination of the previous patterns. Application of phosphoric acid in concentrations of 30% to 37% for 30 seconds results in the formation of calcium phosphate with loss of adamantine structure.

In the dentin, the adhesion is more complex because it is a porous, wet tissue, composed of hydroxyapatite particles in a collagen protein matrix, which compromises adhesion. It has a low intermolecular force and low surface energy. It shows in its composition of 50% to 70% inorganic matter, 20% to 30% organic matter and 10% to 20% humidity. The dentinal tubules increase their diameter and density in relation to their proximity to the pulp. The water content in dentin is lower on the surface and higher in the vicinity of the dental pulp. In addition, dentin is a tissue subjected to numerous changes that appear with age physiologically, entering a process of aging, with an increase in the thickness of dentin and reduction of its permeability caused by sclerosis and caries. For this reason, adhesion is achieved in this tissue due to hybridization and integration processes [23]. This micromechanical retention is manifested in the enamel as an interlocking of the resinous material in the porosities created by the acid etching, while at the level of the dentin an entanglement of the “resin” with the exposed collagen occurs. Although some adhesives have a mild pH and cannot expose all the collagen, hybridization can be achieved by ionic bonds between related components such as the acid monomers of the adhesive and the calcium of hydroxyapatite [24,25,26,27].

The adhesion seeks to generate a strong union, retaining the restorative material or the cementing agent, minimizing microfiltration, marginal pigmentation, secondary caries and reduction of stress by contraction. Another goal of the adhesion is to generate less invasive procedures that avoid tissue wear with retentive purposes [24,26,28].

### 1.1. Classification of Adhesive Systems

The classifications of the adhesive systems have been numerous: By generations, by number of clinical steps and by modes of action [29].

The classification by generations determined by the dental industry, refers to the order in which the adhesive systems were developed according to their complexity. Each generation has sought to reduce the number of containers involved in the process, also simplifying the number of steps [23].

Adhesive systems have also been classified according to the clinical steps used during the process, which reflects their essential mode of use, leaving aside their historical development [23].

### 1.2. Etch–Rinse Adhesive Systems

This system consists of etching the enamel and dentin with 35–37% orthophosphoric acid, which once it has achieved its objective is eliminated with a rigorous rinse [28], followed by the in situ polymerization of the adhesive by own effects of capillarity, it flows in the porosities created in the enamel forming macro tags of entangled resin around the enamel prisms, and in complement, the formation of micro tags that penetrate the cores of the enamel prisms, contributing effectively to the retention of the material.

In the dentinal tissue the effect of the acid generates a network of micro pores in the collagen, where hydroxyapatite is almost absent. Therefore, the adhesion depends on the hybridization or infiltration of the adhesive within the exposed collagen mesh, and in this context the “chemical” adhesion is questioned since it depends on the union of the functional monomers with the hydroxyapatite [30]. This system of etching and washing adhesives can be used in either 2- or 3-step systems.

In the 3-step system, the acid etching, the application of the primer and the application of the adhesive itself, are carried out separately. In the 2-step system, acid etching is performed separately, simplifying the impregnation of the primer and the adhesive in one step.

The main thing in the etching and rinse method is the application of the first or hydrophilic component of the adhesive that usually dissolves in water or volatile solvents such as acetone and ethanol, tertiary butane also being used. The use of these so-called “water hunters” facilitate the elimination of water on the dentin surface accompanied by the exposure of the collagen mesh ready to receive the hydrophobic portion of the adhesive. Therefore, it is important to consider the solvent in function. For example, when using the technique of “wet bonding”, it is necessary to use an adhesive based on acetone, whereas on the contrary in the technique “dry bonding”, an ethanol-based adhesive is recommended [26,30,31].

### 1.3. Self-Etch Adhesives

Due to the simplification of the operative times offered by this technique, it is probably one of the most promising and also involves reducing the sensitivity of the process; that is, a lower risk of making mistakes by not needing a phase of etching and washing.

An important advantage in this method is that the infiltration of the adhesive system occurs simultaneously with the self-etching process, such that there is a reduction in the risk of discrepancies between the two processes [28]. These can be applied in one or two steps, since the retentive capacity is in the partial elimination of the smear generated during the cavity preparation, incorporating in the adhesion process the crystals and residual fragments of the hydroxyapatite, so the retention achievements can be attributed to three main factors:

(1) A cleaning effect on the surface, chemical and mechanical, which starts with the removal of the periphery of the enamel prisms with regular wear of the surface. During the removal of the acid, the dissolved salts are also removed in the created porosities;
(2) A partial demineralization effect that facilitates the crosslinking of the adhesive by means of the generated micro-porosities and the collagen network (hybridization); and
(3) The chemical interaction of the weak acid with the residual hydroxyapatite, giving rise to a network of collagen coated by hydroxyapatite with exposed fibrils, describing a double adhesion both mechanical and chemical. This reaction occurs for example with the system Clearfil SE where the monomer 10 MDP (10-methacryloyloxydecyl dihydrogen phosphate) interacts with the hydroxyapatite of the barrel [32].

However, there is also a questioning of the effects on the surplus of the solvent, that is, whether or not it may affect the integrity of the bond created, or a greater risk of microfiltration, or even the possibility of affecting the infiltrated monomers, in the same way the structure created is more hydrophilic and therefore more prone to degradation by hydrolysis [30,33,34,35].

The self-etching effect is achieved by the incorporation of acidic monomers that fulfill the function of non-selective etching, carboxylic acid groups or phosphates, and depending on the aggressiveness of the acid in function, they can be divided into strong self-etching adhesives and soft self-etching adhesives. The first ones usually have a pH of 1 that gives rise to a considerably deep demineralization effect. If its effect at the enamel level is compared, it is the same as if it were treated with phosphoric acid, and at the dentinal tissue residual hydroxyapatite is almost totally eliminated. This means that these strong self-etching adhesives act technically equal to those of engraving and washing, presenting low values of bond strength. The soft self-etching adhesive systems have a pH of 2 instead, and their dentine demineralization effect is superficial, approximately of 1 μm. With this effect, it is possible to conserve hydroxyapatite residues adhered to the collagen and at the same time create a satisfactory amount of micropores for the micromechanical crosslinking of the adhesive. Although it is true that the thickness of the hybrid layer is reduced in comparison with the strong acids of the etching and washing system, the preservation of hydroxyapatite can allow the reception of additional chemical bonds [24,30,32,35,36].

### 1.4. Causes of Failure of Indirect Restorations

Among the causes that can be mentioned are the following: Position of the cervical contour of the restoration [3,37] and presence of cavitary bases such as the glass ionomer, since this coating contributes to the deterioration of the interface generating fractures. Another factor is the number of steps of adhesive process used. The 3-step is the standard in the adhesive technique and considering the reduced protocol adhesives as a risk factor that reduces the long-term durability of ceramic restorations [3,38]. Additionally, secondary caries, associated with deterioration of the cement and the adhesive layer in addition to the applied cyclic load, can be another failure cause [39]. Excessive drying of the dentine generating a decrease in intrinsic humidity and digital pressure with low intensity can also be considered a risk factor [14], so too extension of the lesion [40,41] and contamination with saliva during the adhesion process [42]. Other associated factors are the minimal thickness of the ceramic restoration, the degree of translucency and the color of the cement since the time of curing by the manufacturer does not always achieve the objective of polymerizing [17]. The masticatory load may influence the physical and chemical properties of the resin–ceramic bond, i.e., this cyclic load, which is approximately 30,000 times per year [43], may propagate throughout the material, resulting in the weakening of the restoration [4,16]. The degree of conversion and the monomer type of simplified systems are factors that affect the mechanical strength of cements [44]. Another issue is the microfiltration that facilitates the passage of bacteria, oral fluids, molecules and ions [45]. Another risk factor is the presence of cavitary bases, since this coating contributes to the deterioration of the interface, generating fractures, delamination and cracks in the interface associated with the microfiltration process, compromising the durability of the bond. A study conducted by Cervino et al. demonstrated the appearance of the interface formed between adhesive agents and the MTA (Mineral Trioxide Aggregate). In this study, the use of adhesive etching and washing systems was considered not very efficient since the presence of water as part of the MTA makes it incompatible with the hydrophobic behavior of the resinous materials, observing a gap of 13.42 microns between the MTA-adhesive-resins. This contrasts with the fact that when using a self-adhesive resinous material, a gap of 6.71 microns was observed [46].

### 1.5. Effect of Water

There are several mechanisms that contribute to the degradation of the dentine resin interface, among which are the hydrophilic nature of the monomers used, the concentration of water required for the creation of ionic bonds in self-etching systems, and the wet technique applied. For this reason, water plays an important role in the hydrolytic degradation of the adhesive polymers, constituting 10% of the weight of the dentine, and when it is treated with 37% phosphoric acid it generates a demineralization of the hydroxyapatite crystals from 5 to 10 μm, leaving “non-adhered” water as a substitution, representing 75% to 79% of the total, while the remaining 21% to 25% is non-free water [47]. Under ideal conditions the adhesive system must replace the free water product of the demineralization, thus avoiding compromising the dentine adhesive interface. As mentioned, the all-in-one or self-etching systems have a higher rate of low mechanical resistance, which may be associated with the fact that they behave as permeable membranes. They allow the passage of water after its polymerization, due to its high content of hydrophilic materials and irregular hydrophobic matrix. It must be added that the same transudation of the dentinal tubules generates “water trees” along the length of the dentine adhesive interface. This absorption generates a plasticization of the adhesive system resulting in the reduction of the microtensile strength when the structure is subjected to loading. This effect can be considered even more serious when using weak or soft self-adhesives that can form a hybrid layer of 0.5 to 1 μm [48], which occurs, for example, with methacrylate 2-hydroxyethyl (HEMA) that has a decrease in its properties after 24 h after the absorption of water and unreacted monomers. This high ionic concentration facilitates the adhesive dentin interface to be hypertonic in relation to the surrounding dentinal tissue and the differences in the osmotic gradient induces the movement of water from the dentine to the interface. In addition, the water content in the adhesive influences the change of bisphenol to diglycidyl methacrylate (BisGMA) [25]. Another important factor is to consider the components of the adhesive systems and to know their affinity for water [48]. It has not been possible to determine if the free water is completely replaced by the resinous component, however in this situation the solvents can have direct influence, since the monomers are dissolved in organic solvents to facilitate the diffusion of the monomer in the collagen matrix and allow the elimination of water during evaporation of the solvent [49]. For all of the above, it was considered relevant to carry out a systematic review of the adhesive systems used in the cementing of indirect restorations, based on the available scientific evidence and analyzing characteristics of the bond strength of these systems to the dental tissues, which may help the clinician in his daily practice to base decisions on.

## 2. Materials and Methods

We analyzed the MEDLINE digital databases (PubMed), and the Cochrane Library with a search strategy based on the combination of MeSH (Medical Subject Headings) keywords. This systematic review used the PRISMA guide (Preferred Reporting Items for Systematic Reviews and Meta-Analysis). The PICO system (Population, Intervention, Comparation, Outcomes) was also used as an eligibility criterion in the selection of articles [50]. The selection of studies was made between the period of July to October 2018. The date of the last search was made on 29 October 2018.

**Population:** Scientific articles on adhesive systems used in indirect restorations.
**Intervention:** Testing of bond strength, resistance to shear and microtraction of the adhesive systems used in indirect restorations.
**Comparison:** Different types of adhesive systems, engraving and rinsing, self-etching and universal systems.
**Outcomes:** bond strength in MPa of the different adhesive systems evaluated.

### 2.1. Inclusion Criteria

In vitro studies.

Articles that focus on the evaluation of the bonding strength of adhesive systems.

Articles in English published between the period 2012 to 2018.

Studies in indirect restorations.

Available full text articles.

### 2.2. Exclusion Criteria

Studies applied in deciduous dentition.

Adhesion strength studies applied in radicular posts.

Studies that evaluate the bond strength in direct restorations.

Studies that evaluate the bond strength after surface pretreatments.

In vivo studies.

### 2.3. Search Terms

The search terms were derived from the previous reading of scientific articles, used as a guide for the writing of this systematic review, so the keywords to be used were the following: Indirect restorations, bond strength, self-etch, etch and rinse, adhesive system, and resin cements. The different search strategies used are described below:

{(“bond strength”) AND (“indirect restorations”)}, {((“bond strength”) AND (“self-etch”) AND (“adhesives”[MeSH] OR adhesive system))}, {((“bond strength”) AND (“etch”) AND (rinse) AND (“adhesives”[MeSH] OR adhesive system))}, {(“adhesives” [MeSH]) OR “adhesives” AND (“indirect restorations”).

### 2.4. Data Collection

The articles were analyzed according to the title, year of publication and design of the article. They were selected under three contexts, the title, the abstract and the full text. The first phase was carried out by obtaining the titles of the articles of interest from the selected database, where titles that differed clearly from the eligibility criteria were directly excluded. The second phase was focused on reading and analyzing the summaries that passed the first filter. If during the reading of the summary it was verified that the article did not have relation with the selection criteria it was discarded. Finally, in the third phase, a critical reading was made of the articles, in their full text, to verify that the selected studies met the selection criteria established for the preparation of the present systematic review.

## 3. Results

### 3.1. Search Strategy

**Figure 1:**
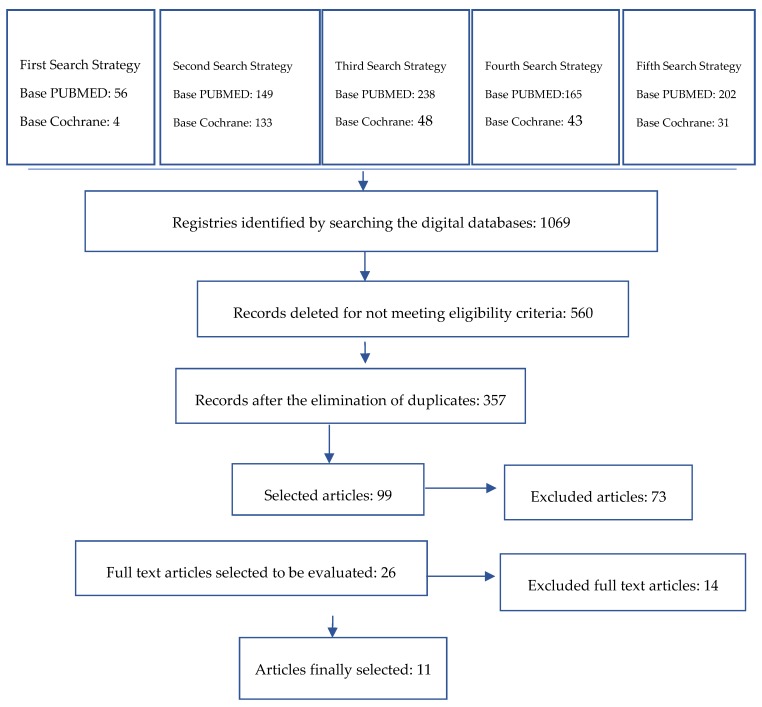
Literature review flowchart.

A total of 1069 potentially relevant articles were identified. Duplicate articles and those that did not meet the aforementioned exclusion criteria were eliminated. Of the remaining studies, 26 were subjected to a detailed evaluation. Finally, 11 articles about enamel/dentin bond strength were selected. The complete process is described in the flow chart (Figure 1). The bonding strength of adhesive etching and rinse systems and self-adhesive systems applied in different clinical steps were evaluated. The details of the author, the adhesive system used as well as the strength of union to the tissue are described in the Table 7.

#### 3.1.1. Study Quality Assessment

The bias of the studies finally analyzed in this review was evaluated using methodological risk factors specific to the study. Parameters were used such as random distribution of the samples, teeth free of caries, control group, samples with similar size, evaluation of the failure mode, description of the coefficient of variation, calculation of the sample size and the presence of at least one blind examiner. When the evaluation parameter was present or absent, the word Yes and No were assigned respectively. The articles were classified as having a high risk of bias when at least 3 positive parameters were obtained; of medium risk when 4 were obtained; and of low risk of bias when the studies presented at least 5 risk factors (Table 1).

#### 3.1.2. Bond Strength

Bond strength has been evaluated according to the dental substrate, and the adhesive system according to its classification by the number of steps used in the adhesive protocol (Table 2, Table 3, Table 4, Table 5, Table 6 and Table 7).

## 4. Discussion

Indirect restorations with the trend of CAD/CAM systems are frequently used in the daily clinic. Therefore, the knowledge of adhesive systems and the cementation protocol is an important step that must be well-founded for the final decision of the clinician. Several parameters are quantified to evaluate the adhesive strength of these systems. Bond strength is a quantitative parameter in order to determine the effectiveness of the adhesive systems in enamel and dentine. Several studies did not show results in enamel. After this systematic review, it was observed that the conventional adhesive systems of two and three steps had greater bond strength, independently of the study test and the trademark used. In this study, due to the large number of samples, methods and trademarks it has not been possible to give an accumulated quantitative value. However, it was confirmed that conventional systems continue to show a better performance. It is important to establish differences between the methodology to test the bond strength. Studies showed high values for shear bond strength until 445 MPa [21]. Microtensile bond strength tests show values in general in order from 6 to 67 MPa. Adhesion values were different between enamel and dentine because of the differences in the organic component. Moreover, the number of steps is an important factor to evaluate the adhesive systems. There was even an improvement in the performance of self-conditioning systems with the previous application of conventional etching and rinse systems [9,10,15,18] (Table 2, Table 3, Table 4, Table 5 and Table 6). Pamato and collaborators carried out a study to evaluate the different hybridization techniques and their influence on the resistance of self-adhesive resinous cements. This study confirmed that the dentin hybridization treatment with different adhesive systems presents diverse behaviors associated with both cement and etching techniques. The study samples were divided into six groups according to the conditioning applied in dentin. Of these, the Adper Scotchbond 3-step adhesive system had the highest bond strength (16 MPa) while the U200 control group had the lowest value (11.19 MPa) [51]. Simplified systems have been used to reduce operating times by eliminating the pretreatment of the substrate. However the quality of the interface formed is directly associated with the infiltration of the monomers into the tissue, which is generally shallow due to the high viscosity exhibited despite its low initial pH [52]. Adper Scotchbond showed a significantly higher adhesion strength of all the groups evaluated. On the other hand, Opti Bond presented a reduced value despite being a 3-step system. This could be explained by the presence of ethanol as a solvent in this system that can generate greater dehydration of the collagen matrix [27]. Likewise, Pekperdahci et al. in their research studied the shear bond strength after a thermocycling process in cementing systems and self-adhesive systems to which they were given different surface conditioning. The control group in this case was RelyX ARC + Adper Single Bond 2, a system of total etching that presented the highest values of union (370.07 MPa) (Table 2). While the simplified system presented the lowest values (RelyX Unicem + Adper-Prompt L-pop, 77.06 MPa) [21]. With respect to this, both Pamato and Pekperdahci agree that the self-adhesive systems are not capable of forming a true hybrid layer, which is corroborated with the images obtained through the analysis under the stereomicroscope carried out in Pekperdahci´s study where they showed the formation of an irregular, shallow and deficient hybrid layer. It was also found that the pre-treatment of the surfaces and the application of an adhesive can improve the bond strength of the self-adhesive cements [51]. Previous etching can favor the creation of a more humid surface and bring on a better ionization of the acidic monomers and better linking of the polymers in the simplified protocols [14]. With similar results, Chávez and colleagues carried out a study comparing the dentin adhesion strength of four self-adhesive cements compared with a control group of self-adhesive cement plus etching. With the results, it was concluded that the traditional protocol (acid etching and application of the adhesive system followed by the cement) had a better performance with a higher value of shear strength compared to the simplified protocols. The RelyX ARC + Single Bond 2 control group presented the highest binding values (15.52 MPa) [52]. The behavior of the simplified protocols is associated with its low demineralization capacity that reduces the infiltration in the dentin tissue. Although this cement has a high initial pH, the increase in its viscosity can influence the poor formation of the hybrid layer. In addition, in order to achieve its objective, these materials must infiltrate the dentin in a short time, which may be possible under conditions of wettability of the dentin [53]. According to the manufacturers, the capacity of the self-adhesive is due to the presence of acidic monomers in the formula of the cementing agents, which once fulfilled its function should recover to a neutral pH. It has been shown for cements such as MaxCem that the pH remained at 3.6 which ends up being detrimental to the bond strength in the dentin [54].

Vaz et al. also evaluated the microtensile bond strength in indirect restorations cemented with different polymerization cements and a different cementation protocol after 42 h and 30 days of storage in water. After 24 h of storage in water, the highest bonding values were for Adper Single Bond 2 + RelyX ARC with 40.8 MPa and the lowest values were for C&B, a self-polymerization cement + All Bond 2 with 19.5 MPa (Table 2). After thirty days the values were 44.2 and 24.5 MPa, respectively. In the evaluation of interfacial micromorphology, the All Bond 2 group presented an irregular and granular hybrid layer in comparison with the Adper Single Bond 2 group [55]. These results may be due to the fact that acetone-based adhesives such as All Bond 2 have a lower monomer-solvent ratio, which means that they require a greater number of applications or layers to achieve a more uniform bonding interface [29] (in this study two layers were applied). In the case of Adper single Bond 2, the formation of resin tags can be associated with its wettability, since it presents ethanol as a solvent. As for the increase in the bonding strength of C&B, it can be associated with the fact that the polymerization process was completed with the passage of time, thus reinforcing its adhesive properties. Like the previous studies [21,51,56], the behavior of the RelyX ARC + Adper Single Bond 2 group is associated with the capacity of the adhesive system used to form a hybrid layer of greater uniformity and depth that is achieved with total etching systems, and added to this the dual polymerization of cement [57]. Under the same concept Ozturk and colleagues evaluated the shear bond strength of ceramic laminates in different substrates and with different conventional adhesive systems. Study groups were divided according to the substrates. Results showed low bonding values for the dentin and Rely X Veneer + Adper scotch bond group (5.42 MPa), and the group with the highest bond strength was for the Syntac Primer enamel group (Syntac Adhesive + Variolink Veneer, 24.76 MPa). It was concluded that the dental substrate had a high influence on the bond strength, even more than the adhesive system that can be used, suggesting that the edges of indirect restorations should be in enamel given the greater reliability of adhesion [57]. Roperto et al. evaluated the microtensile strength of ceramic compounds adhered to the dentin using three different adhesive strategies. Of the samples evaluated, Primer and Bond NT + Calibra presented the highest values of union (17.68 MPa), while Panavia F2.0 + Clearfil SE Bond presented 12.22 MPa, and the control group Smart Cem presented 6.48 MPa [58]. With these results, this study has determined that the best strategy for ceramic restorations continues to be the 3-step system. The Clearfil SE group showed acceptable values and its behavior may be associated with 10 MDP, a component that has a good chemical interaction with the hydroxyapatite present in the dentinal smear layer, allowing a greater long-term bond stability [59]. Moreover, the simplified system SmartCem2 evaluated in this study showed low adhesion values, supporting the fact that these systems behave as permeable membranes more prone to hydrolytic degradation, thus reducing the bonding force [48,59]. In accordance with this finding, Skupien et al. found higher bonding values for Scotchbond Multi-Purpose (20.29 MPa) 3-step systems and Adper Single Bond 2 (17.68 MPa) 2-step etching and rinsing systems. The results of this study showed no major differences between these systems. However, the control group with RelyX U100 showed low values of union of 9.69 MPa, also associated with the poor infiltration capacity of the cement in the unconditioned tissue [60].

Lorenzoni et al. evaluated the bond strength of resin cements to dentine with different adhesive systems. Of the samples evaluated All-Bond + Duo-link presented the lowest binding values (6.16 MPa). On the other hand, Adper Single Bond 2 + Duo-link presented the highest values (14.5 MPa) [61]. Different adhesive techniques show different performance values in the dentine. Peumans concluded in his study that the disadvantage of the etching and washing systems are attributed to the sensitivity of the technique and to the presence of ethanol as a solvent that in some cases can generate excessive tissue dehydration. Another factor that may explain these results is the presence of polyalkenoic acid in a large part of the simplified systems of 3 M ESPE, since the high molecular weight of this compound may compromise the interdiffusion of the adhesive in the tissue [60,62]. On the other hand, Bacchi and collaborators conducted a study comparing different adhesive techniques and the influence of intrapulpal pressure after cementation. It was evidenced that the bond strength was greater for the conventional Adper Single Bond 2 + RelyX ARC system without the influence of pulp pressure. After three months of simulation of the pulp pressure, adhesion strength diminished, possibly associated with open dentinal tubules, and acid-etching product and the presence of residual monomers were verified. Another possibility is the presence of collapsed dentinal tubules that were not infiltrated by the adhesive. After the simulation, the group ED Primer (self-etching) + Panavia F2.0 presented an improvement in resistance, which was associated with the conservation of the smear layer inside the ducts that could contribute to a reduction in dentinal permeability. However, in the absence of a significant difference, it is concluded that conventional cements together with an adhesive etching and washing system are considered the standard for cementing indirect restorations [63]. Likewise, Alexandre and colleagues carried out a similar study where they evaluated the effects of simulated pulp pressure on adhesion strength in resin-dentin interfaces produced by different adhesion strategies after 12 months. While it is true that the initial values are high for the etching and rinse system and 2-step Adper Single Bond 2 + RelyX ARC (67.9 MPa), under the influence of pressure pulp this value declined significantly (18.6 MPa) [64]. Like the previous study, this behavior is attributed to the presence of water that was not eliminated during the process. Likewise, certain hydrophilic monomers such as HEMA can attract more water, damaging the mechanical properties of the cements and the stability of the hybrid layer formed. In the case of simplified systems, pH regulation results in water release favoring the behavior of a more hydrophilic cement that improves the wettability of dentine. This procedure is important, since the release of hydrogen ions allows the demineralization of the smear layer. Moreover, this release allows the change of a hydrophilic to hydrophobic behavior that maintains the stability of the bond formed [52,65]. It is concluded that in the absence of pulp pressure the etching and rinsing systems maintain a good behavior and high adhesion strength values. However, in the presence of this factor, the values decline drastically, keeping self-recorded systems more stable. It must be clarified that this is the only study in this review that favors the use of self-etching systems [64].

Some in vitro studies show that the extra step of preconditioning with phosphoric acid, in simplified protocols, improves the behavior at the dentin in bond strength, marginal adaptation and sealing [66,67]. For this reason, selective acid etching has been suggested as an additional step for self-adhesive cements in order to improve adhesion to the enamel by additionally creating micro-retentions. Selective etching in vitro studies have shown a significant improvement in adhesion bond strength [68]. With respect to this, Baader et al. carried out a study comparing the behavior of ceramic partial restorations, cemented with different adhesive techniques with and without previous selective etching, using self-adhesive cements. After 6.5 years of follow-up, an accumulated success rate of 82.1% and 59.8% was determined, which allows determining that the selective etching at the enamel level significantly improves the clinical longevity of the restoration. Main causes of failure in the first group were the fracture of the restorations. However, when analyzing the marginal seal there were no significant differences between the two cementing techniques [14]. The dental hypersensitivity was also associated with selective acid etching especially during first days after cementing, which can be related to the contact of the acid with dentin, in such a way that the elimination of smear layer may increase the risk of postoperative hypersensitivity [14,69,70]. Peumans et al. evaluated the clinical behavior of cemented ceramic inlays and onlays with self-adhesive systems prior to the application of an etching and rinsing system, and concluded in this regard that this methodology can be recommended for the fixation of indirect ceramic restorations and after four years of follow-up have found no clinically significant difference in the application of selective acid etching prior to cementation at the enamel level [71]. Aguiar et al. evaluated the ultrastructure by means of electron microscopy at the dentin–cement–resin interface formed by both self-adhesive and conventional cements. When evaluating RelyX ARC/Adper Scotchbond it was evidenced according to the microscopy, the formation of a true hybrid layer of high and uniform density with the formation of long resin tags of approximately 20 to 35 nm. On the other hand, when evaluating simplified systems, the formation of resin tags was not evident, and they even showed gaps in the interface. This variability can be associated with the chemical composition of the materials, the substrate and the pretreatment method used. In the case of resinous cements that require pretreatment, there is a true process of hybridization that generates a micro mechanic interlock, which represents the retention of indirect restorations, whereas in self-adhesive systems the interaction with the substrate is produced by the chemical reaction of the functional monomers such as 10 MDP (10-methacryloxydecyl dihydrogen phosphate) (Clearfil SA and DC), and the phosphoric acid monomer (RelyX Unicem) [56]. Aguiar et al. evaluated the tensile strength after the effects of cyclic loads and permanence in saliva with similar samples. It was demonstrated in this study that both the self-adhesive cements and the conventional ones had a high tensile strength, the same one that remained with similar values after two years of cyclic loads. Nevertheless, the group of conventional cements, and used with an adhesive system of a single step (Clearfil Esthetic-10 MDP 10-methacryloxydecyl dihydrogen phosphate), showed a decrease in adhesion strength [72]. The problem with simplified adhesive systems is that their acidic monomers consume tertiary amines present in some cements, resulting in incomplete polymerization, reflecting a reduction in bond strength [73].

Regarding universal adhesives, Passia and colleagues evaluated in their study the tensile strength of different systems of universal adhesives used for cementation of ceramics reinforced with lithium disilicate. Their samples were divided into 4 groups consisting of different universal adhesives and resin cements. After the study it was concluded that Monobond Plus showed greater strength of union to the ceramic even after 150 days of storage in water, which suggests that not all the universal systems are applicable for the adhesion of ceramics. [11]. Likewise, Yoshiara and colleagues studied the effectiveness of coupling agents such as silane as a component of universal adhesive systems. A universal Scotchbond silane adhesive system (9.4 MPa) and a universal silane-free adhesive system such as Clearfil S3 Bond ND Quick (9.9 MPa) were used. Significantly higher bond strength was recorded when the Clearfil Porcelain bond activator was freshly mixed with the universal silane-free adhesive (27.3 MPa). Clinically, the separated silane or silane freshly mixed with the adhesive is recommended for the adhesion of ceramic restorations [7]. Nikolaus and colleagues conducted a study in which the influence of various surface treatment methods on the adhesion effectiveness of universal adhesives was evaluated. It was observed that the silane included in the universal adhesive may not be effective in the resin ceramic bond, since the protocol that included one more step (silanization) presented a better behavior with respect to the adhesion strength (independently of the acid etching). In addition, it was determined that acid etching is considered an effective method to eliminate contaminants present and favor the formation of an effective surface for the application of a universal adhesive [42]. On the other hand, Park and colleagues evaluated the influence of different surface treatments on the bond strength to nano-ceramic resin restorations, using a universal adhesive. It was found that the resin cement can be affected by the surface treatment and also by the application of the adhesive material. It was proved that air abrasion and the Rocatec system were very effective in increasing the bond strength of the cement to the ceramics processed in the laboratory (7.64 MPa and 7.41 MPa, respectively), when it was associated with the application of an adhesive system universal. However, these methods can generate a large loss of material so its use must be controlled. Etching with hydrofluoric acid forms a rough and superficial texture creating a honeycomb topography on the surface of the ceramic, ideal for a micro mechanical bond. However, its effect is less compared to the methods mentioned above. Another point of interest was that the need for the use of an adhesive system was confirmed to further increase the strength of the union of nano-ceramic resins and cement [20]. Even Stawarczyk reported the effectiveness of repairing a nano ceramic resin with a universal adhesive system plus a direct resin, although the phosphoric acid monomers work better than an adhesive based exclusively on methacrylic monomers. This behavior is explained by the presence of dihydrogen phosphate (MDP), present in the universal adhesive (Single Bond Universal) [74]. In this sense, it could be concluded that the ability of the adhesive monomers to intertwine with the collagen fibrils depends on several factors: The conditioning of the tissue, the priming steps of the same and the wetting characteristics, the chemical composition of the adhesive systems, the hydrophobic or hydrophilic behavior and the properties of the monomers.

The level of bias of the studies reviewed in this article implies the requirement to make better methodological designs to analyze the adhesive systems used in the cementing of indirect restorations. Likewise, although it is true that the use of in vitro studies is valid, more randomized clinical studies are required to observe the in vivo behavior of the adhesive systems used in a longer term, to determine their clinical longevity (Table 1).

With the development of nanotechnology and nanophase materials, adhesives have also presented modifications within their structure through polymeric nanocomposites with improved mechanical and physical properties. Azad in his study determined that the incorporation of reinforcing nanoparticles positively influences the mechanical properties and bond strength of adhesives to dentin [75]. In agreement with this author, Belli mentions that the incorporation of nanoparticles in self-etching adhesives promotes the strengthening of the adhesive and the adhesive interface. However, he points out that one of the drawbacks of nano-fillers is that they can substantially increase the viscosity of the adhesive and allow the absorption of water in the adhesive layer [76]. Due to a greater tendency to perform minimally invasive restorative procedures, restorative materials have been developed, such as adhesives that have calcium phosphates in their composition, designed with the aim of improving the remineralization of dentine. The incorporation of α-TCP nanoclusters (tricalcium phosphate) in adhesive resins may improve the strength of the junction and may be a promising strategy to achieve therapeutic remineralization at the interface of compound dentin [77]. Modified cyanoacrylates are also found in nano-filled materials, which at concentrations below 20% by weight, provide nanocomposites with improved physical and mechanical properties. A determining factor in the effectiveness of nanoparticles in improving the properties of the polymer matrix is its dispersion quality in the matrices. Adhesives with this type of nanostructure also showed lower water absorption and greater hydrolytic stability. It is concluded that adhesives based on modified cyanoacrylates can be considered promising candidates for dental applications [78]. On the other hand, silica particles as nano-filler material have been shown to increase adhesion resistance, since they act as cross-links that promote bond strength to enamel and dentin. In this sense, nano-sized fillings lead to a total permeabilization of the junction [79]. Despite the promising results of these new versions, even more study is required to guarantee the stability of the components.

## 5. Conclusions

The results obtained are presented based on the selected articles with heterogeneous data, variability of study specimens and evaluation materials. However, this review of the literature exposes a broader and more detailed knowledge, where it can be assured that the etching and rinsing system is still the system of choice. The most evaluated adhesive systems are Adper Scotchbond, Adper Single Bond and Single Bond Universal. Only one study recommended a simplified system, instead of a conventional system, considering the pulp pressure as an influencing factor and its effect of deterioration in the bonding strength of conventional systems. According to this review of the literature, the 3-step system was the most effective due to its lower risk of hydrolytic degradation at the interface level. Unfortunately, it is a highly sensitive technique, which is why more humidity control is suggested depending on the detailed components by the manufacturer.

The self-etched adhesive systems reduce the time spent in clinical practice. However, at the interface they behave as permeable membranes, which facilitates the passage of fluids from oral environment to dentin and vice versa (dentine–intraoral environment), being more susceptible to degradation. Furthermore, its use is limited when using dual and self-curing cements, as its components can interfere with the polymerization process. They are also prone to form a discontinuous, irregular, and shallow hybrid layer associated with low wettability, viscosity of the system, and low infiltration into the dental tissues. More clinical studies with a low level of methodological bias that analyze the adhesive systems and their clinical protocol for cementing indirect restorations are required.

## Figures and Tables

**Table 1:** Risk bias factor.

Study	Teeth Randomization	Caries-Free Teeth	Control Group	Samples with Similar Dimensions	Evaluation of Failure Mode	Description of Coefficient of Variation	Sample Size Calculation	Blinding of the Examiner	Risk of Bias
Pamato	Yes	Yes	Yes	Yes	Yes	No	No	No	Medium
Aguiar	Yes	yes	No	Yes	Yes	No	No	No	Medium
Ozturk	Yes	Yes	No	Yes	Yes	No	No	No	Medium
Skupien	Yes	Yes	No	Yes	Yes	Yes	No	Yes	Low
Lorenzoni	Yes	Yes	No	Yes	Yes	No	No	No	Medium
Pekperdahci	Yes	Yes	Yes	Yes	Yes	No	No	No	Medium
Chávez	Yes	Yes	Yes	Yes	Yes	Yes	No	No	Low
Vaz	Yes	No	Yes	Yes	Yes	No	No	No	High
Bacchi	Yes	Yes	Yes	Yes	Yes	No	No	No	Low
Alexandre	Yes	Yes	No	Yes	Yes	No	No	No	Medium
Roperto	Yes	Yes	Yes	Yes	No	No	No	No	Medium

**Table 2:** Bond strength of etch and rinse 3-step systems (MPa).

Author	3-Step Adhesive System	Cement	Enamel Bond Strength (MPa)	Dentin Bond Strength (MPa)
Pamato	Optibond	U200	Data Not Available	13.3
Pamato	Adper Scotchbond	U200	Data Not Available	16
Aguiar	Adper Scotchbond	Rely X ARC	Data Not Available	17.1
Ozturk	Adper Scotchbond	RelyX Veneer	22.46	5.42
Ozturk	Heliobond Syntac	Variolink II	23.64	13.78
Ozturk	Heliobond Syntac	Variolink Veneer	24.76	13.84
Skupien	Adper Scotchbond	RelyX ARC	Data Not Available	20.29
Lorenzoni	Adper Scotchbond	Duo Link	Data Not Available	11.6
Lorenzoni	Optibond	Duo Link	Data Not Available	12.29
Lorenzoni	All Bond 3	Duo Link	Data Not Available	6.16

**Table 3:** Bond strength of etch and rinse 2-step systems (MPa).

Author	2-Step Adhesive System	Cement	Enamel Bond Strength (MPa)	Dentin Bond Strength (MPa)
Pamato	Adper Single Bond 2	U200	Data Not Available	15.79
Pekperdahci	Adper Single Bond 2	RelyX ARC	Data Not Available	370.07 ∗
Pekperdahci	Adper Single Bond 2	RelyX Unicem	Data Not Available	96.56 ∗
Chavez	Adper Single Bond 2	RelyX ARC	Data Not Available	15.52
Vaz	All Bond 2	C&B Cement	Data Not Available	19.5
Vaz	Adper Single Bond 2	RelyX ARC	Data Not Available	40.8
Bacchi	Adper Single Bond 2	RelyX ARC	Data Not Available	25
Alexandre	Adper Single Bond 2	RelyX ARC	Data Not Available	34.8
Skupien	Adper single Bond 2	RelyX ARC	Data Not Available	17.68
Roperto	Primer and Bond NT	Calibra	Data Not Available	17.68
Lorenzoni	Adper Single Bond 2	Duo Link	Data Not Available	14.5

∗ Shear bond strength test.

**Table 4:** Bond strength of 1-step self-etch adhesive systems.

Author	1-Step Adhesive System	Cement	Enamel Bond Strength (MPa)	Dentine Bond Strength (MPa)
Pamato	Bond Force	U200	Data Not Available	15.0
Pekperdahci	Adper Prompt	Rely X Unicem	Data Not Available	77.06 ∗
Aguiar	Clearfil DC Bond	Clearfil Esthetic Cement	Data Not Available	13.3
Bacchi	ED Primer	Panavia F2.0	Data Not Available	15
Lorenzoni	Bond Force	Duo Link	Data Not Available	9.4

∗ Shear bond strength test.

**Table 5:** Bond strength of 2-step self-etch adhesive systems.

Author	2-Step System	Cement	Enamel Bond Strength (MPa)	Dentine Bond Strength (MPa)
Alexandre	Panavia 21	Panavia F2.0	Data Not Available	7.8
Alexandre	Clearfil SE Bond	Panavia F2.0	Data Not Available	38.0
Roperto	Clearfil SE Bond	Panavia F2.0	Data Not Available	12.22

**Table 6:** Bond strength of universal adhesive systems (MPa).

Author	Universal Adhesive System	Cement	Enamel Bond Strength (MPa)	Dentine Bond Strength (MPa)
Pamato	Single Bond Universal	U200	Data Not Available	12.6
Lorenzoni	Single Bond Universal	Duo Link	Data Not Available	8.3

**Table 7:** Study characteristics.

Author	Test	Aging	Storage	Highest Value/Lowest Value (MPa)
Saulo Pamato	Microshear bond strength	______________	_____________	Adper Single Bond 2: 16<break/>U 200: 11.190
Pekperdahci	Microshear bond strength	Water 5 °C/55 °C for 10,000 cycles	Distilled water at 37 °C for two days	ARC Single Bond 2: 370.07 Relyx Unicem/Adper Prompt 77.06
Chávez	Microshear bond strength	_____________	Distilled water and kept at a constant temperature of 37 °C	Relyx ARC Single Bond 2: 15.52 Set PP 3.17
Ozturk	Microshear bond strength	Thermocycled between 5 °C and 55 °C in Deionized water for 5000 cycles.	Distilled water for 24 h at 37 °C	Variolink Venner/Heliobond: 24.76 (enamel) RelyX Veneer/Adper Scottchbond: 5.42 (dentin)
Lorenzoni	Microshear bond strength	______________	________________	Adper Single Bond 2/Duo Link: 14.5 All Bondl/Duo Link: 6.16
Aguiar	Microtensile bond strength	Mechanical load cycling, submitted to 50,000 cycles	Artificial saliva for 1 day	Relyx Unicem: 21.3 Clearfil Esthetic Cement/Clearfil DC Bond: 13.3
Vaz	Microtensile bond strength	______________	37 °C and 100% relative humidity	Relyx ARC/Adper Single Bond 2: 40.8 C&B Cement/All-Bond 2: 19.5
Bacchi	Microtensile bond strength	Pulpal pressure for 3 months	20 cm distilled water	Relyx ARC/Adper single bond 2: 25 U200: 8
Alexandre	Microtensile bond strength	Pulpal pressure for 24 h	Distilled water	Clearfil SE Bond/Panavia F: 38.0 Panavia 21/Panavia F2.0: 7.8
Roperto	Microtensile bond strength	_______________	Water for 24 h at 37 °C	Primer and Bond NT/Calibra: 17.68 Smart Cem: 6.48
Skupien	Microtensile bond strength		Distilled water at 37 °C for 24 h	Adper Scotchbond/Relyx ARC: 20.29 U100: 9.69
